# Dimensions Underlying Measures of Disability, Personal Factors, and Health Status in Cervical Radiculopathy: A Cross-Sectional Study

**DOI:** 10.1097/MD.0000000000000999

**Published:** 2015-06-19

**Authors:** Marie Halvorsen, Marie Kierkegaard, Karin Harms-Ringdahl, Anneli Peolsson, Åsa Dedering

**Affiliations:** From Division of Physiotherapy, Department of Neurobiology, Care Sciences and Society, Karolinska Institutet, Stockholm, Sweden (MH, MK, KH-R, AD); Department of Physiotherapy, Karolinska University Hospital, Stockholm, Sweden (MH, MK, KH-R, AD); and Department of Medical and Health Sciences, Physiotherapy, Faculty of Health Sciences, Linköping University, Linköping, Sweden (AP).

## Abstract

This cross-sectional study sought to identify dimensions underlying measures of impairment, disability, personal factors, and health status in patients with cervical radiculopathy.

One hundred twenty-four patients with magnetic resonance imaging-verified cervical radiculopathy, attending a neurosurgery clinic in Sweden, participated. Data from clinical tests and questionnaires on disability, personal factors, and health status were used in a principal-component analysis (PCA) with oblique rotation.

The PCA supported a 3-component model including 14 variables from clinical tests and questionnaires, accounting for 73% of the cumulative percentage. The first component, *pain and disability*, explained 56%. The second component, *health, fear-avoidance beliefs, kinesiophobia, and self-efficacy*, explained 9.2%. The third component including *anxiety*, *depression*, and *catastrophizing* explained 7.6%. The strongest-loading variables of each dimension were “present neck pain intensity,” “fear avoidance,” and “anxiety.”

The three underlying dimensions identified and labeled *Pain and functioning*, *Health, beliefs, and kinesiophobia*, and *Mood state and catastrophizing* captured aspects of importance for cervical radiculopathy. Since the variables “present neck pain intensity,” “fear avoidance,” and “anxiety” had the strongest loading in each of the three dimensions; it may be important to include them in a reduced multidimensional measurement set in cervical radiculopathy.

## INTRODUCTION

Cervical radiculopathy (CR) forms an important subgroup of neck disorders. The annual incidence is approximately 85 cases per 100,000 in the population.^1^ Characteristically, CR is associated with symptoms of neck-, shoulder- and upper-limb pain, upper-limb paresthesia and weakness, and reflex changes.^2^ Patients with combined neck and arm pain report lower health status than those with neck pain alone.^3^ The major cause of CR is degenerative disease of the spine. Spondylosis, disc protrusion, or both are reportedly responsible for CR in 68% of patients, and disc protrusion alone in 22%.^1,4^ CR affects both sexes, with a peak incidence in the age group 40 to 60 years.^1^

Patients with CR are often initially treated conservatively^5,6^ which is also suggested as first-line treatment.^7,8^ Patients whose pain does not naturally resolve may require surgical intervention, especially if they have significant extremity or myotomal weakness or severe pain; or if the conservative treatment is unsuccessful.^9^

The development of chronic pain may involve an interaction between the injury, the experience of pain and psychological factors such as anxiety, depression, fear-avoidance beliefs, kinesiophobia, and catastrophizing.^10^ In patients with neck- and shoulder pain psychosocial factors are important predictors for development of chronic pain with low levels of pain catastrophizing being favorable for outcome.^11,12^ However, it is unclear which factors contribute to the development of chronic pain and disability in CR patients.

In the few descriptions of nonsurgically treated patients with CR, they are presented in terms of magnetic-resonance imaging findings and body functions such as radiating pain, sensory and motor impairments, neck motion range, and muscle endurance.^13^ Less is known about this disability in the form of activity limitations and participation restrictions; or about personal factors such as anxiety, depression, fear avoidance behavior, kinesiophobia, self-efficacy, and coping strategies. To form a complete picture and capture important aspects of patients with CR, a broad measurement battery including clinical tests and questionnaires is needed.^14^ Such a battery could involve a risk that measures might overlap. Additionally, a battery could be a burden for the patient in pain due to the time and effort needed. Hence the importance of exploring whether there are common underlying dimensions that explain the CR patient's characteristics. It is also important to identify variables as key measures for inclusion in a measurement battery for CR. The present aim was therefore to identify dimensions underlying measures of disability, personal factors, and health status in CR patients.

## MATERIALS AND METHODS

### Design and Participants

This cross-sectional study comprised analyses of clinical examinations, test results, and questionnaire answers in patients with CR recruited from a university-hospital neurosurgery clinic in Sweden. Inclusion criteria were CR and pathology of relevance for CR verified with magnetic-resonance imaging, and positive results on the Spurling sign test^15^ and/or a cervical extension test. Exclusion criteria were former cervical fracture, luxation, and surgery; spinal infection and malignity; known drug abuse; diagnosed psychiatric disorders; other diseases or disorders that could interfere with participation in treatment or measurements, and unfamiliarity with the Swedish language. A total of 124 patients (mean age 48, range 20–75 years), 59 men and 65 women, fulfilled the criteria and were included. All patients had the diagnosis cervical disc disorder with radiculopathy (M50.1) classified in the International Classification of Diseases, 10th version (ICD-10). Sixteen of the 124 patients had 1 additional diagnosis and 4 had 2 additional diagnoses; systemic lupus erythematosus with organ or system involvement (M32.1), deforming dorsopathy, unspecified (M43.9), other spondylosis cervical spondylosis without myelopathy or radiculopathy (M47.8), spondylosis, unspecified (M47.9), spinal stenosis (M48.0), other specified spondylopathies ossification of posterior longitudinal ligament (M48.8), lumbar and other intervertebral disc disorders (M51.0), cervicalgia (M54.2), nerve root and plexus compressions in other dorsopathies (G55.3). None of the patients were in a structured rehabilitation program but were screened for enrolment in a randomized clinical trial to evaluate exercise treatment. Oral and written information was given and all patients provided signed informed consent before enrolment. The study was approved by the Regional Ethical Review board in Stockholm and procedures were conducted in accordance with the Helsinki Declaration. The study follows the STROBE guidelines for reporting observational studies.

### Clinical Examinations and Tests

A standardized neurological assessment protocol was performed by the same test leader (author and physiotherapist M.H.). It included bilateral examination of sensory function (light touch and pin prick) in dermatomes C4–C8; motor function (manual muscle testing) of key muscles in myotomes C4–T1; and reflexes for C5–C7 (biceps, triceps, and brachioradialis). An abnormal sensory and/or motor function in at least one of the tested dermatomes and myotomes, respectively, or an abnormal response in at least one of the tested reflexes, was classified as impairment.

Neutral head posture in sitting was measured as described by Engh et al^16^ using a goniometer (Vinkelmätare Brodin, Medema, Sweden) measuring the angle between the two goniometer arms in degrees. The centre of the goniometer was placed at the orifice of the external ear from where one arm hung vertically and the other pointed to the C7–T1 spinal motion segment.

Active range of motion of neck flexion, extension, lateral flexion, and rotation in the sitting position was measured using a cervical-range-of-motion instrument (Performance Attainment Associates, Roseville, MN).

Neck-muscle endurance time was measured in seconds during submaximal, static, dorsal, and ventral neck muscle contractions performed in prone and supine positions, respectively, as previously described.^17,18^

Balance function was evaluated in the walking-in-figure-of-eight test^19–21^ and the sharpened Romberg test performed with eyes closed (tandem standing with right or left foot behind).^21,22^ Participants were allowed 3 trials. If no steps in the figure-of-eight test were incorrect in the first or second trial, no further trials were performed. The Romberg test was measured in seconds and terminated if the participants opened his or her eyes, or reached the maximum value of 30 seconds. If this was reached in the first or the second trial no third trial was performed. The mean value of the trials was calculated for each test.

Weight (kilograms) and height (meters) were measured and body mass index (kg/m^2^) was calculated for descriptive purposes.

### Questionnaires

A set of study-specific and standardized questionnaires was sent to participants the week before the clinical assessments, to be filled out at home and handed in at the assessment. There were questions on sociodemographic and background data, for example, social- and work status, smoking habits, pain history, and symptoms and signs. The set also included standardized questionnaires on disability, personal factors, and health status, as described below.

Pain duration was reported in months and dichotomized into subacute (<3 months) and chronic (≥3 months) neck pain. Present neck pain intensity was assessed on a visual analogue scale (VAS) ranging from 0 (no) to 100 mm (worst imaginable). Average neck pain, arm pain, and headache intensity were calculated as the mean of VAS ratings (mm) of current, best, and worst during the previous week. Average dizziness intensity was calculated as the mean VAS ratings (mm) of dizziness at rest, during motion, and perceived unsteadiness problems.

The Neck Disability Index (NDI)^23,24^ was used to assess the effect of neck pain on functioning and disability. The NDI consists of 10 items rated on a 6-point scale, ranging from 0 to 5. Results are summed to a total score that can be expressed as a percentage. A higher percentage score indicates greater disability.

The Dizziness Handicap Inventory (DHI)^25^ was used to assess self-perceived disability imposed by dizziness. Its 25 items are rated on a 3-point scale (0, 2, and 4), and summed to a total score. A higher score indicates more disability. The DHI includes 3 response levels: functional, emotional, and physical, but in this study only the total sum score was used.

The Pain Catastrophizing Scale (PCS)^26^ was used to assess catastrophic thoughts or feelings in relation to painful experiences. The PCS consists of 13 items which are rated on a 5-point scale from 0 to 4, and summed to a total score. Higher scores indicate higher levels of pain catastrophizing.

The Hospital Anxiety and Depression Scale (HADS)^27^ was used for evaluating anxiety and depression. Its 14 items (7 anxiety and 7 depression) are rated on a 4-point scale (range 0–3). A higher score indicates a higher level of anxiety or depression.

The Self-Efficacy Scale (SES)^28^ for assessing patients’ perceived confidence in performing different activities in spite of pain consists of 20 items rated on an 11-point scale, ranging from 0 to 10, and summed to a total score. Higher scores reflect greater self-efficacy.

The Fear Avoidance Beliefs Questionnaire (FABQ)^29,30^ is a self-reported inventory that focuses specifically on patients’ beliefs about how physical activity and work affect their pain. Its 16 items are rated on a 7-point scale, ranging from 0 to 6, and summed to a total score. Higher scores indicate higher levels of fear-avoidance beliefs.

The Tampa Scale of Kinesiophobia (TSK)^31,32^ was used to assess the patients’ current pain-related fear of movement/(re)injury. The TSK has 17 items rated on a 4-point scale from 1 to 4. After inverting the score for items 4, 8, 12, and 16, a total sum-score for all items is calculated. Higher scores indicate higher levels of kinesiophobia.

The Coping Strategies Questionnaire (CSQ)^33^ was used to assess patients’ use of cognitive and behavioral strategies to cope with pain. The CSQ consists of 50 items which are rated on a 7-point scale, ranging from 0 to 6, and 48 items are summed to a total score. The 2 additional items are reported separately and evaluate the patient's self-perceived control over pain (CSQ-COP) and ability to decrease pain (CSQ-ADP).

The Exercise Self-Efficacy Scale (ESES)^34,35^ was used to assess patients’ confidence in performing an exercise program despite potential barriers. The 6 ESES items are rated on a 10-point scale, ranging from 1 to 10, and summed to a total score. Higher scores indicate greater confidence.

The short version of the International Physical Activity Questionnaire (IPAQ-short) was used to measure patients’ self-reported physical activity during the previous 7 days.^36^ The IPAQ-short consists of questions about time spent in sitting, walking, moderate-intensity physical activity and in vigorous intensity physical activity to estimate total weekly physical activity expressed as MET-hours per week (MET = metabolic equivalent, 1 MET = resting energy expenditure).

Physical activity levels during the previous summer and winter half-years were assessed with the Saltin-Grimby Physical Activity Level Scale.^37^ This six-graded scale ranges from hardly any physical activity to heavy or very heavy exercise regularly and several times a week.

The EuroQol 5D (EQ-5D) consisting of the EQ-5D Index and EQ-5D VAS was used as a measure of health status.^38^ The EQ-5D consists of 5 dimensions (mobility, self-care, usual activities, pain/discomfort, and anxiety/depression). These are rated on 3 levels (no problems, some problems, or extreme problems). The answers are converted to an index score using the time-trade-off value set. Negative index scores were set to zero and possible scores ranged from 0 to 1 (full health).^39^ The EQ-5D VAS consists of a 200 mm vertical line, scored 0 (worst imaginable) to 100 (best imaginable), on which the respondent marks his/her own perceived health state today.

### Data Analysis

Descriptive statistics were used to present mean and standard deviation (SD), minimum and maximum values, frequency, and percentage.

Missing data in the questionnaires were handled as follows: for missing items less than 30%, an imputation value was calculated, that is, the mean value of the nonmissing item. Questionnaires with more than 30% missing data were excluded from the analyses.

A principal-component analysis (PCA) with oblique rotation was carried out to identify underlying dimensions. Oblique rotation was chosen as it was assumed that components would be correlated.^40^ Variables were chosen from clinical examinations and tests, and questionnaires (Table 1). The EQ-5D VAS was, however, excluded as the EQ-5D Index represented a health-status measure. Likewise, the 2 individual items CSQ-COP and CSQ-ADP were excluded since the CSQ total sum was used. Before the PCA, data were checked for normality and outliers. Six nonnormally distributed variables were square-root transformed to normality (Table 1) and 1 was excluded as it could not be so transformed (figure-of-eight test). There were no problems with outliers.

**TABLE 1:**
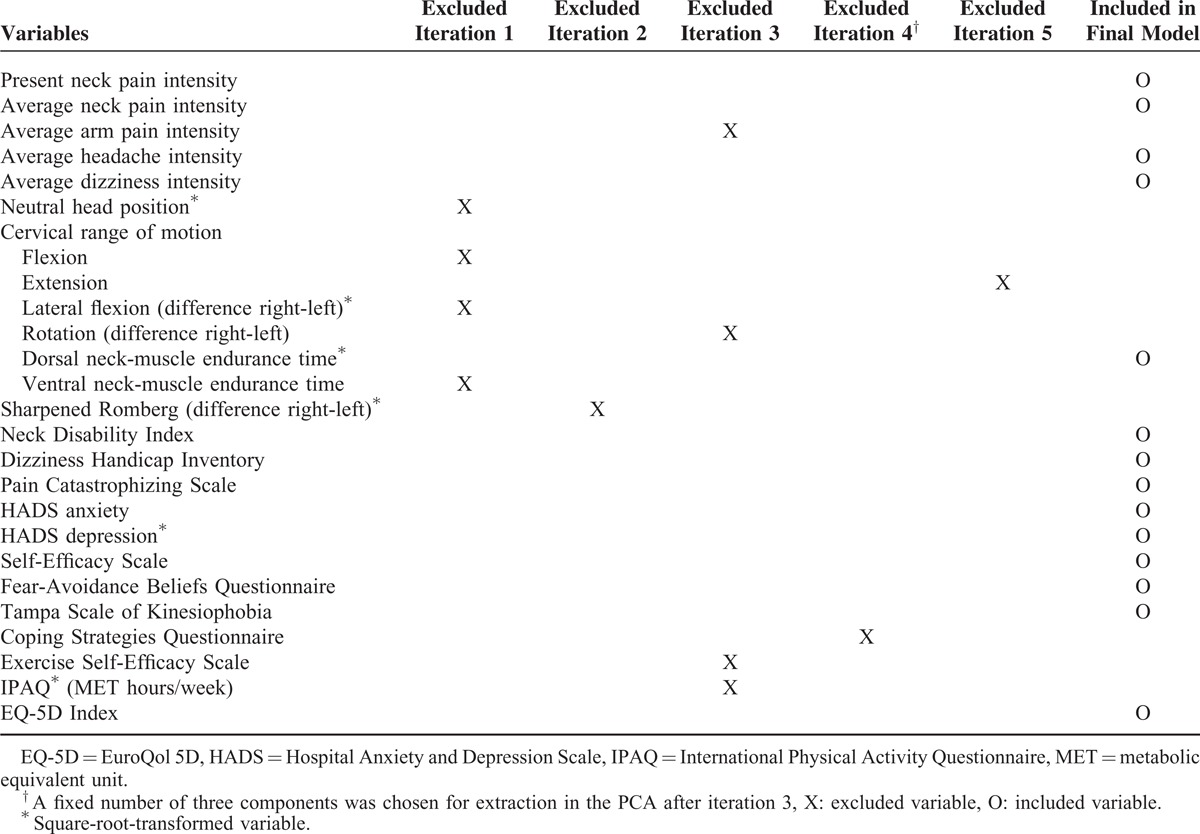
Variables Used in the Principal-Component Analysis (PCA) With Oblique Rotation

Iterations of the PCA were conducted until variables fulfilled the criteria to be included, that is, until the individual Kaiser-Meyer-Olkin (KMO) value and/or communalities were over 0.50. The number of components to be extracted was based on the Kaiser criterion of eigenvalues equal to or exceeding one and on inspection of the Scree plot.^40,41^ These indicated that 3 components should be extracted. The variables excluded from iteration and those included in the final model are presented in Table 1. The correlation matrix was used to assess whether there was a problem of multicollinearity, that is, correlation coefficients exceeding r = 0.90 or too low, and the determinant of the matrix should be >0.00001. The reproduced matrix giving a summary of how many residuals (differences between observed correlations and those based on the model) with an absolute value >0.05 was used to assess the fit of the final model. The percentage of nonredundant residuals with absolute values over 0.05 should be <50%. The KMO measure of sampling adequacy and Bartlett's test of sphericity were used as measures of appropriateness of the PCA. A KMO value between 0.80 and 0.90 was interpreted as very good and Bartlett's test of sphericity as significant, that is, *P* < 0.05.^40^

The pattern matrix containing information about the unique contribution of a variable to the component and the structure matrix taking account of the relationship between components were used to present the results of the PCA with oblique rotation. Component loadings were interpreted as suggested by Tabachnick and Fidell,^41^ that is, loadings over 0.71 = excellent, loadings between 0.63 and 0.70 = very good, between 0.55 and 0.62 = good, between 0.45 and 0.54 = fair, and loadings below 0.32 = poor. The component correlation matrix showing correlation coefficients between components was used to evaluate independence of the underlying dimensions.

All the analyses were performed using the soft ware Statistical packages for the social sciences for Windows (release 22).

## RESULTS

Patients’ characteristics and descriptive data from clinical examinations, tests, and questionnaires are presented in Tables 2–4. A majority of the participants were classified as having chronic neck and/or arm pain, in many experienced daily. Headache and dizziness were less frequent. Most participants worked full or part time. Approximately half were physically inactive during both summer and winter, and the mean body mass index value was 26 kg/m^2^ indicating that some half were overweight. Sensory impairments were more common than motor impairments. Ventral neck-muscle endurance time was on average much shorter than that of dorsal neck muscles. The mean VAS values for present and average pain intensities ranged from 27 to 43 mm, which can be considered mild.^42^

**TABLE 2:**
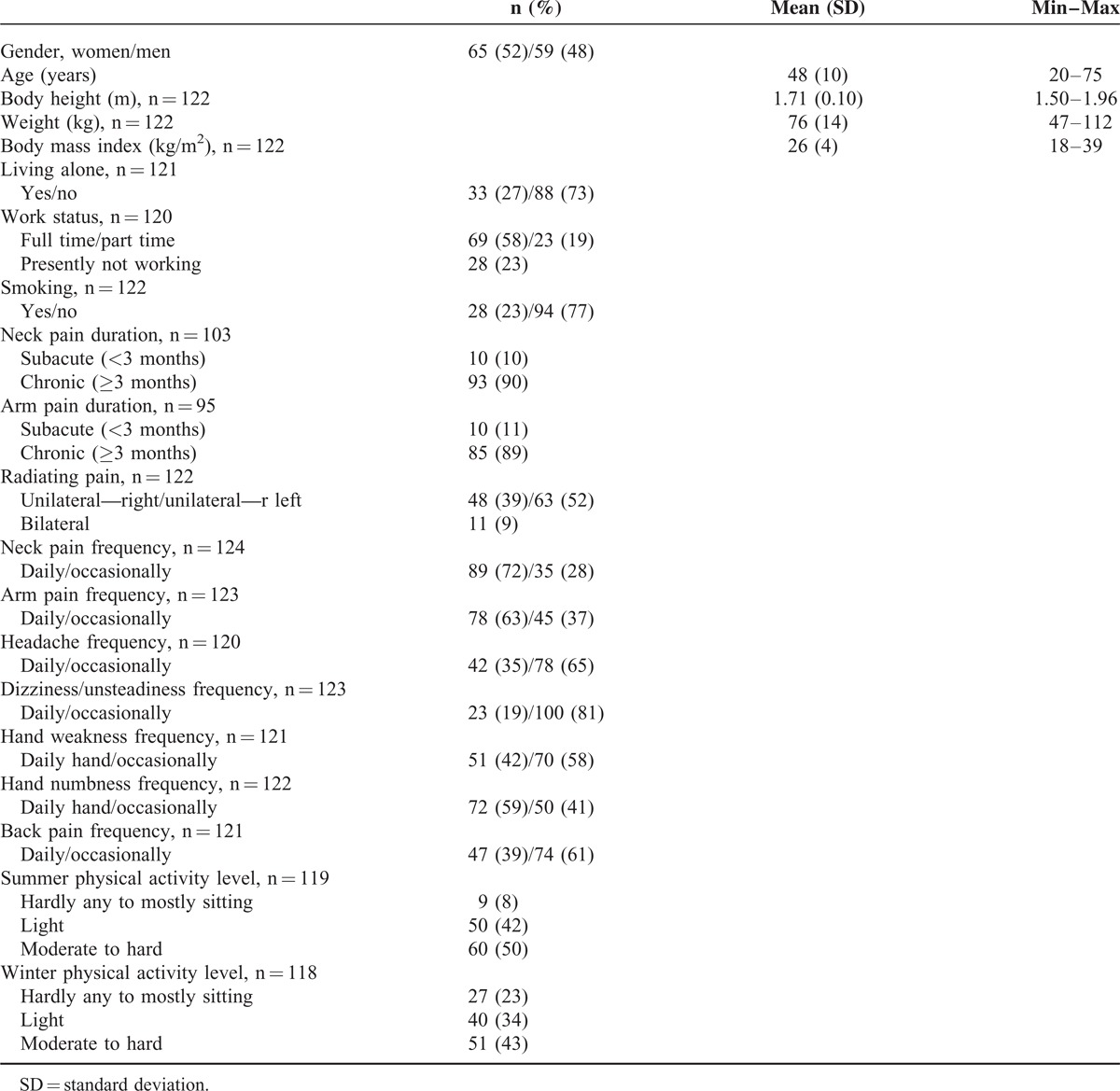
Characteristics of Patients With Cervical Radiculopathy (n = 124)

**TABLE 3:**
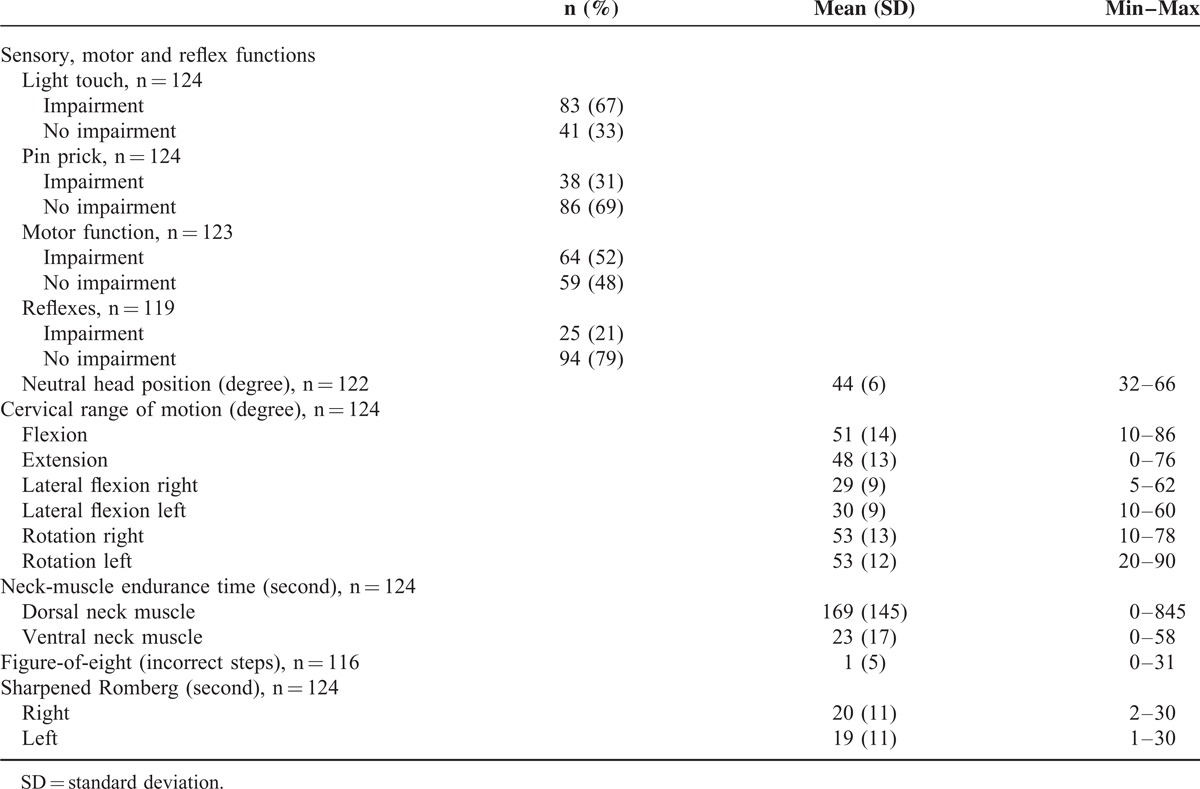
Results From Clinical Examinations and Tests for Patients With Cervical Radiculopathy (n = 124)

**TABLE 4:**
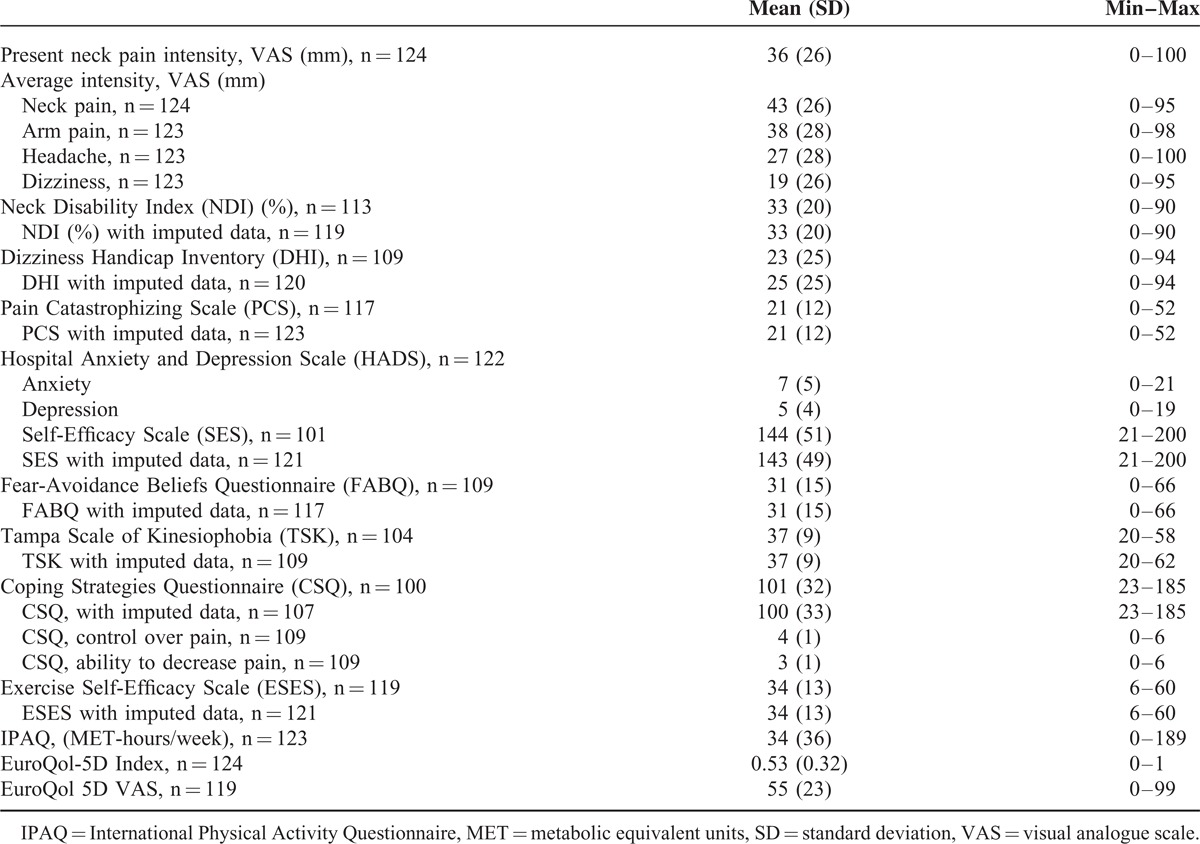
Results From Questionnaires for Patients With Cervical Radiculopathy (n = 124)

### Underlying Dimensions of Disability, Personal Factors, and Health Status

The final model was a 3-component solution, indicated by both eigenvalues and the Scree plot, accounting for 73% of the total variance. Included were 14 of the originally 20 variables (Table 1) with data from 93 participants. The correlation matrix indicated no problem of multicollinearity, that is, correlation coefficients between variables were over 0.20 and below 0.90, and the determinant of the matrix was >0.00001. The percentage of nonredundant residuals with absolute values over 0.05 was 45%, supporting the fit of the model. Communalities after extraction were all above 0.60, except one which was 0.56. All individual KMO values exceeded 0.80, the overall KMO measure of sampling adequacy was 0.89 and Bartlett's test of sphericity was highly significant: Chi-square (91) = 972, *P* < 0.001.

The 3-component solution of the PCA is presented in Table 5. Component loadings after oblique rotation, that is, regression coefficients, for each variable onto each component are presented in the pattern matrix (Table 5). Oblique rotation also gives another set of component loadings, that is, the correlation coefficients between each variable and component, presented in the structure matrix (Table 5).

**TABLE 5:**
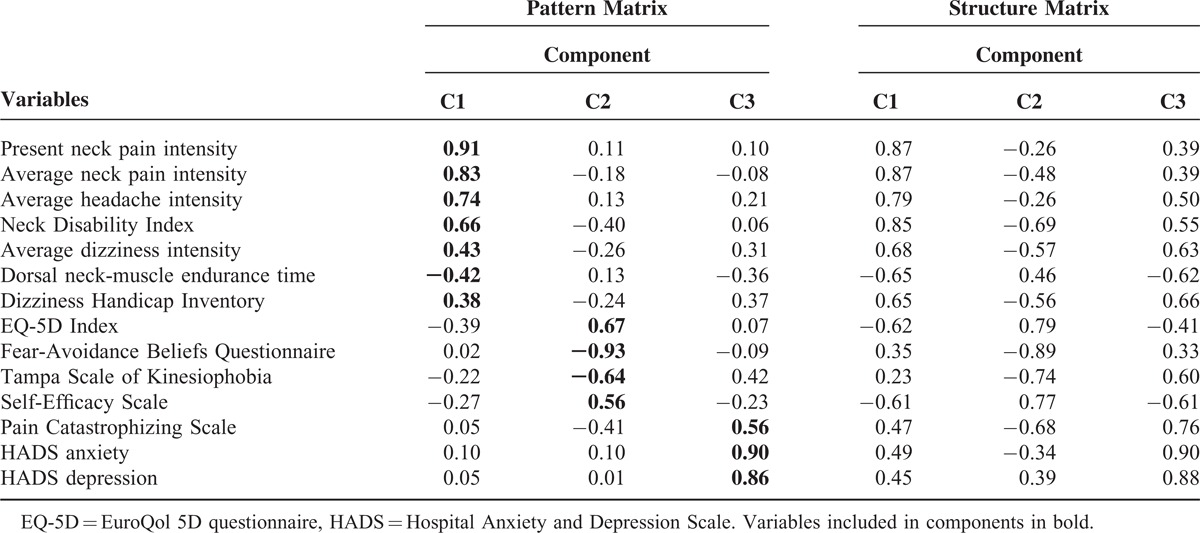
Results of the Principal-Component Analysis (PCA) With Oblique Rotation Showing the 3-Component Model (C1–C3) and Component Loadings in the Pattern Matrix and in the Structure Matrix (n = 93)

The first component (C1) explained 56% of the variance and had 4 variables with component loadings over 0.63, that is, in the range very good to excellent. These variables concerned pain and functioning in relation to pain. Thus, the component was labeled *Pain and functioning*. The other three variables, concerning dizziness and dorsal-neck-muscle endurance, had component loadings just below the fair level. The variables “present neck pain intensity” and “average neck pain intensity” provided most of the information in C1 (Table 5).

The second component (C2), explaining 9.2% of the variance, had 3 of 4 variables with component loadings in the range very good to excellent, those with the highest loadings being the FABQ and the EQ-5D Index. This component was labeled *Health, beliefs, and kinesiophobia* (Table 5).

The third component (C3) explained 7.6% of the variance and had 2 variables with component loadings interpreted as excellent and one with a loading considered good. The strongest-loaded variable was HADS anxiety, closely followed by HADS depression. The component was labeled *Mood state and catastrophizing* (Table 5).

The component correlation matrix showed that correlation coefficients between components were close-to-moderate, indicating that an oblique-rotated solution was to be preferred and that the underlying dimensions could be interrelated. Correlation coefficients were for *Pain and functioning* and *Health, beliefs, and kinesiophobia* −0.40, for *Pain and functioning* and *Mood state and catastrophizing* 0.47, and for *Health, beliefs, and kinesiophobia* and *Mood state and catastrophizing* −0.49.

## DISCUSSION

This study gives a thorough description of nonsurgical patients with CR from the perspective of impairments, disability, personal factors, and health status. Our main finding was the 3-component solution with 14 variables that clustered into the underlying dimensions *Pain and functioning*, *Health, beliefs, and kinesiophobia*, and *Mood state and catastrophizing*.

*Pain and functioning* appeared to be the most important component of the model as it accounted for most of the variance. Our findings are supported by 2 earlier studies reporting strong relations between neck-specific disability and pain intensity.^19,43^ Other studies have reported a positive relation between instruments measuring pain and physical functioning (r = 0.53–0.70).^23,44,45^

That fear-avoidance beliefs and self-efficacy loaded on the same component, *Health, beliefs, and kinesiophobia*, is supported by previously reported findings of relations between measures of such variables.^46,47^

Although the variable PSC had its highest loading in the component *Mood state and catastrophizing*, the structure matrix indicated that it was also related to the component *Health, beliefs, and kinesiophobia*. Earlier studies show that pain-catastrophizing thoughts could predict pain-related fear which could lead to avoidance of movement.^48–50^ Fear-avoidance beliefs in patients with neck pain are important since they may predict disability and return to work.^51^

We decided to group the variables related to dizziness (average dizziness intensity and DHI) in the component *Pain and functioning* as they had their highest component loadings there. However, these variables seemed to be related to the other 2 components as shown by the moderate correlations presented in the structure matrix. Dizziness and unsteadiness in patients with CR is sparsely evaluated,^52,53^ but has shown a larger impact on perceived health-related-quality-of-life than pain intensity levels in individuals with mechanical neck pain, including those with degenerative changes in the cervical spine.^54^ An explanation of balance disturbances in patients with cervical pain might be a change in cervical structure^55^ with altered proprioception^56^ leading to motor control deficits,^57^ which might affect neck-muscle endurance. Experimental studies on healthy people have shown neck muscle fatigue to have an impact on balance and posture.^58,59^ Limited neck-muscle endurance was previously shown to be a significant impairment and strongly related to pain in patients with CR.^18,60,61^ Interestingly, dorsal muscle endurance time was moderately correlated both to the component *Pain and functioning* and to *Mood state and catastrophizing*. Patients with high levels of neck pain are more depressed than people without.^62^ However, the relation between endurance time, depression, and pain catastrophizing ought to be studied further since it might contribute to worse health status in patients with CR.

Although the criteria for performing a PCA were fulfilled, there are issues to be discussed. A common rule for sample size is that there should be 10–15 participants per variable. On the other hand, the literature suggests that 5–10 participants for each variable included may be adequate for robust results if all communalities are above 0.6.^40,41^ We chose to use only total scores for each questionnaire in order to reduce the number of variables. As there were missing data, imputations were made when possible to maximize the sample size. There were 93 valid cases for the current set of variables, which should thus be acceptable for performing the PCA as the communalities in our model were consistently above the 0.6 level.

Most variables had high component loadings on 1 component and low on the others. Some variables, however, seemed to be related to more than 1 component. An explanation might be that we used total scores from questionnaires and not scores from possible subscales.

Although our primary focus was to identify underlying dimensions, a PCA is also applicable for data reduction without losing the original information. This is achieved by summarizing many variables into fewer components while trying to explain as much data variance as possible.^63^ There are various ways to choose how many components to retain from a PCA, for example, the choice may be based on eigenvalues, Scree plots, percentage of variance explained, sufficient variables with high loadings in each component and the communalities. Our 3-component model was supported by eigenvalues over 1, high explained variance, high component loadings, and communalities. The variables that provided most information (highest loadings) in each component, respectively, in our PCA model were neck pain, fear-avoidance beliefs, and anxiety. It might be important to always include these when assessing patients with CR. However, with regard to variable loadings and pattern matrix, there are reasons to consider also the variables measuring the impact of pain on functioning and disability, health status, kinesiophobia, and depression, so as to capture a broad picture of patients with CR. Further research is needed for evaluating the importance of these variables as outcome measures in intervention studies.

## CONCLUSION

We identified three underlying dimensions, that is, *Pain and functioning*; *Health, beliefs, and kinesiophobia*; and *Mood state and catastrophizing* in measures of disability, personal factors, and health status in patients with CR. Pain, fear avoidance beliefs, and anxiety provided most information in the components and it is therefore suggested that these variables are important to include in assessments of patients with CR.
